# Systems Biology Approaches for the Prediction of Possible Role of *Chlamydia pneumoniae* Proteins in the Etiology of Lung Cancer

**DOI:** 10.1371/journal.pone.0148530

**Published:** 2016-02-12

**Authors:** Shahanavaj Khan, Ahamad Imran, Abdul Arif Khan, Mohd Abul Kalam, Aws Alshamsan

**Affiliations:** 1 Nanomedicine Research Unit, Department of Pharmaceutics, College of Pharmacy, PO Box 2457, King Saud University, Riyadh 11451, Saudi Arabia; 2 King Abdullah Institute for Nanotechnology, King Saud University, PO Box 2455, Riyadh, 11451, Saudi Arabia; Rush University Medical Center, UNITED STATES

## Abstract

Accumulating evidence has recently supported the association of bacterial infection with the growth and development of cancers, particularly in organs that are constantly exposed to bacteria such as the lungs, colon, cervical cancer etc. Our *in silico* study on the proteome of *Chlamydia pneumoniae* suggests an unprecedented idea of the etiology of lung cancer and have revealed that the infection of *C*. *pneumoniae* is associated with lung cancer development and growth. It is reasonable to assume that *C*. *pneumoniae* transports its proteins within host-intracellular organelles during infection, where they may work with host-cell proteome. The current study was performed for the prediction of nuclear targeting protein of *C*. *pneumoniae* in the host cell using bioinformatics predictors including ExPASy pI/Mw tool, nuclear localization signal (NLS) mapper, balanced sub cellular localization predictor (BaCeILo), and Hum-mPLoc 2.0. We predicted 47/1112 nuclear-targeting proteins of *C*. *pneumoniae* connected with several possible alterations in host replication and transcription during intracellular infection. These nuclear-targeting proteins may direct to competitive interactions of host and *C*. *pneumoniae* proteins with the availability of same substrate and may be involved as etiological agents in the growth and development of lung cancer. These novel findings are expected to access in better understanding of lung cancer etiology and identifying molecular targets for therapy.

## Introduction

Lungs cancer is the most common cause of death worldwide [1]. In 2013, according to CA cancer reports, lung cancer is second most prevalent cancer in US as there were an estimated 159,480 (118,080 males and 110,110 females) deaths from lung cancer and 228,190 (male 87,260 and 72,220) new cases of lung cancer reported [2]. The process of carcinogenesis of lung cancer is still not completely understood. Beside smoking, there are other potential genetic and environmental factors such as exposure to asbestos and radon definite metals, coal smoke, various hormones, and air pollution as well as genetic incompatibility and chronic infections of bacteria and parasites have been connected to lung carcinogenesis including *C*. *Pneumoniae*, [3–6]. The equivocal association of infectious agents in the etiology of cancer has focused the interest of scientists in recent year.

The role of *C*. *pneumoniae* as an infectious carcinogen in lung cancer has been studied since more than 10 years ago [7, 8]. Epidemiological associations indicated that *C*. *pneumoniae* is potentially associated with the growth and development of lungs cells carcinoma [9]. Various studies were then performed to analyze the possible connection between *C*. *pneumoniae* infection and risk of lung cancer, but the results have not been consistent [10]. It is proposed that the *C*. *pneumoniae* acts as a cofactor with other causes for the progression and development of lungs caner [7, 8, 11, 12]. It has been observed that the titers of *C*. *pneumoniae* antibody elevated in lung cancer patients. Among witch, patients with high titers of anti-*C*. *pneumoniae* IgA antibody have ten-time risk of adenocarcinomas and small cell carcinomas of the lung [9]. This possibility is enhanced specifically in male smoker patients with chronic infection of *C*. *pneumoniae* [11]. One more finding have demonstrated an important connection between elevated *Chlamydia Hsp-60* seropositivity and the chance of lung cancer, which may suggest the etiological role of *C*. *pneumoniae* in the growth and development of lung cancer [3].

*C*. *pneumoniae* is a common intracellular respiratory pathogen which requires regulating the host cell for their survival and growth. The following mechanisms have been suggested to elucidate how chronic infection of *C*. *pneumoniae* could enhance the possibility of lung cancer. One potential mechanism is mediated through the generation of reactive oxygen species during inflammation, which may contribute to DNA damage [7]. Moreover, inflammation results in cell injury and subsequent repair that may enhance the rate of cell division. The multiplication of cells will increase the risk of a mutation through a fixed rate of DNA damage, which may lead to cancer [13]. Collecting evidence proposes that immunological events contribute in part in the carcinogenic action of *C*. *pneumoniae*. Earlier *in vitro* studies have in fact shown that TNF-α, IL-1β, IL8, and superoxide oxygen radicals released by alveolar macrophages from healthy persons play a crucial role in lung tissue and DNA damage [14]. *C*. *pneumoniae* is also effective inducer of TNF-α, IL-1β, and IL-6 in host monocytic cells that may potentially contribute in carcinogenesis. In this paper, we are trying to predict the nuclear targeted proteins of *C*. *pneumoniae* due to their potential role in the host cells regulation and involvement in the progression and development of lung cancer.

## Results

### Selection of protein database

We selected TW-183 strain of *C*. *pneumoniae* as it contains highest number (1112) of protein in complete proteome G/11222. However, the rest of the four strains include AR39, LPCoLN, CWL029 and J138 have 1109, 1105, 1052, and 1069 proteins, respectively [15–18].

### Prediction of nuclear localization signal

The cNLS mapper predicted the location of protein in cytoplasm, both nucleus and cytoplasm, partially in nucleus, and localized in the nucleus with cut of value 1–2, 3–5, 7–8, and 8–10, respectively. The results were illustrated in Supplementary data (S1 Table).

### Prediction of subcellular localization in eukaryotic cell organelles

The results of BaCeILo assessment of total protein of *C*. *pneumoniae* summarized in Table 1. The result showed that BaCeILo predicted total cytoplasmic (515), mitochondrial (183), nuclear (98) and Secretory (318) proteins.

**Table 1:** Possible sub-cellular localization of *C*. *pneumoniae* proteins in host cell as per BaCeILo and Hum-mPLoc 2.0.

Type of Protein	Number of Proteins predicted by BaCeILo	Number of proteins by predicted Hum-mPLoc 2.0
Cytoplasm	513	250
Mitochondria	183	196
Nucleus	98	292
Secretory	318	-
Plasma Membrane	-	94
Endoplasmic reticulum	-	63
Golgi apparatus	-	05
Peroxisome	-	11
Microsome	-	01
Lysosome	-	11
Extracell	-	179
Centrosome	-	05
Cytoskeleton	-	01
Unknown		04

### Prediction of subcellular localization in human cell organelles

The results of Hum-mPLoc 2.0 shows in Table 1 and compared with the result of BaCeILo. The result showed that Hum-mPLoc 2.0 predicted total cytoplasmic (250), mitochondrial (196), nuclear (292) plasma membrane 94, endoplasmic reticulum 63, Golgi apparatus 05, peroxisome 11, microsome 01, lysosome 11, extracell 179, centrosome 05, cytoskeleton 01 and unknown (04) proteins.

### Synchronization of BaCeILo predicted proteins with Hum-mPLoc 2.0 predictor

The synchronization results indicated that among the BaCeILo predicted total cytoplasmic (515), mitochondrial (183), nuclear (98) and secretory (318) proteins, not all follow the same prediction results by Hum-mPLoc 2.0 (Table 2). When these BaCeILo proteins further compared with Hum-mPLoc 2.0 results, only 47 proteins were found consistent with BaCeILo results and showed nuclear localization through *in silico* prediction by both computational tools.

**Table 2:** Possible sub-cellular localization prediction of *C*. *pneumoniae* proteins with BaCeILo and their synchronization with Hum-mPLoc 2.0 predictions.

S. No	Intracellular distribution of BaCeILo predicted proteins as per Hum-mPLoc 2.0			
	Cytoplasmic (513)	Nuclear (98)	Mitochondrial (183)	Secretory (318)
1.	Cytoplasm: **167**	Cytoplasm: **13**	Cytoplasm: **32**	Cytoplasm: **38**
2.	Mitochondrion: **111**	Mitochondrion: **18**	Mitochondrion: **47**	Mitochondrion: **19**
3.	Nucleus: **131**	Nucleus: **47**	Nucleus: **42**	Nucleus: **73**
4.	Centrosome: **03**	Centrosome: **0**	Centrosome: **02**	Centrosome
5.	Cytoskeleton: **01**	Cytoskeleton: **0**	Cytoskeleton	Cytoskeleton
6.	Endoplasmic reticulum: **15**	Endoplasmic reticulum: **03**	Endoplasmic reticulum: **12**	Endoplasmic reticulum: **33**
7.	Extracell: **46**	Extracell: **12**	Extracell: **19**	Extracell: **102**
8.	Golgi apparatus: **1**	Golgi apparatus: **0**	Golgi apparatus: **02**	Golgi apparatus: **02**
9.	Lysosome: **2**	Lysosome: **0**	Lysosome: **02**	Lysosome: **07**
10.	Microsome: **1**	Microsome: **0**	Microsome	Microsome
11.	Plasma Membrane: **25**	Plasma Membrane: **05**	Plasma Membrane: **23**	Plasma Membrane: **41**
12.	Peroxisome: **7**	Peroxisome: **0**	Peroxisome: **02**	Peroxisome: **02**
14.	Unknown: **3**	Unknown: **0**	Unknown	Unknown: **01**

These 47 host nuclear-targeted proteins illustrated in Table 3 and arranged as per their NLS cutoff value. Increasing the cutoff value of monopartite NLS are linked with decreased nuclear targeting, whereas the reverse pattern is observed with bipartite NLS values, where the high cutoff value enhances the percentage of protein targeting to nucleus except cutoff value >8 (Table 3). The S1 Table provides details about predicted proteins target to nucleus during *in silico* analysis.

**Table 3:** Computationally prediction of *C*. *pneumoniae* proteins targeting to nucleus of host cell and their relation with all proteins with similar NLS.

NLS	NLS cutoff	Number of proteins targeting to nucleus	Total number of proteins in this range	Percentage
Monopartite	0–3.0	36	981	3.66
	3.0–5.0	5	64	7.81
	5.0–8.0	5	50	10
	>8.0	1	17	5.88
Bipartite	0–3.0	8	274	2.91
	3.0–5.0	17	616	2.75
	5.0–8.0	21	209	10.04
	>8.0	1	13	7.69

Nevertheless, not any accurate relation was found between nuclear targeting protein and molecular weight, but the increased molecular weight consistently increased nuclear targeting except the one range of molecular weight 60–80 kDa, the highest molecular weight proteins (>80 kDa) were observed most targeted proteins to nucleus of host cell (Table 4).

**Table 4:** Computationally prediction of *C*. *pneumoniae* proteins targeting to nucleus of host cell and their relation with proteins with similar molecular weight.

Molecular weight	Number of proteins Targeting to Nucleus	Total number of proteins	Percentage
0–20 kD	9	326	2.76
20–40 kD	16	384	4.16
40–60 kD	13	235	5.53
60–80 kD	1	87	1.14
>80 kD	8	80	10

Furthermore, the value of isoelectric point (pI) did not show any constant pattern for mitochondrial targeting (Table 5).

**Table 5:** Computationally prediction of *C*. *pneumoniae* proteins targeting to nucleus of host cell, and their relation with all proteins with similar pI value.

Range of pI value	Number of proteins targeting to Nucleus	Total number of proteins	Percentage
3.0–5.0	3	106	2.83
5.0–6.0	11	297	3.70
6.0–7.0	10	175	5.71
7.0–8.0	4	79	5.06
8.0–9.0	8	150	5.33
9.0–10	10	215	4.65
10.0–11.0	1	70	1.42
11.0–13.0	0	20	0.0

**Fig 1:**
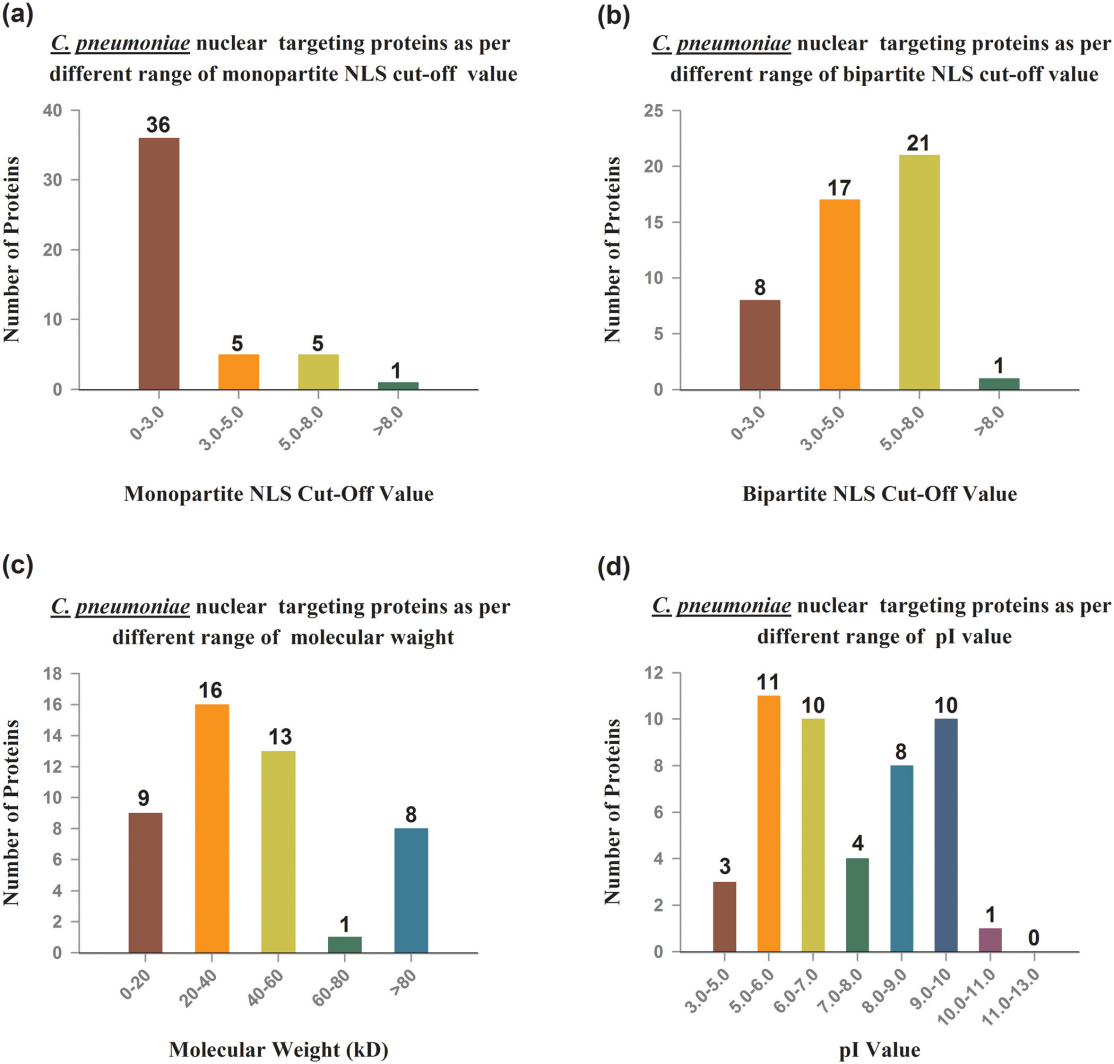
Computationally prediction of *C*. *pneumoniae* proteins targeting to nucleus of host cells and their relation with various parameters.

**Fig 2:**
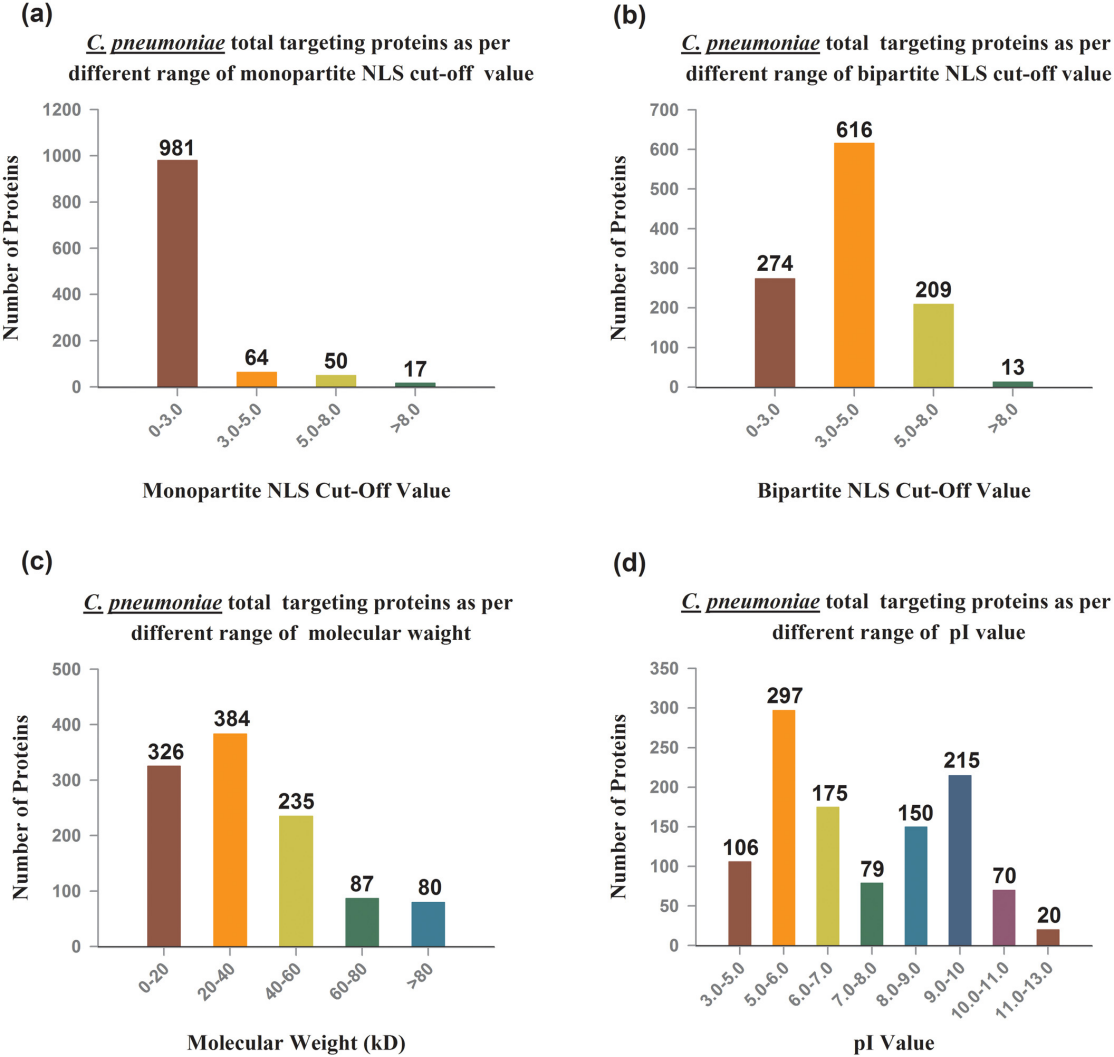
Computationally prediction of whole proteome of *C*. *pneumoniae* proteins (UniProt data base) and their relation with various parameters.

The patterns of *C*. *pneumoniae* protein targeting in host cell nucleus with different parameter shows in Fig 1, whereas the all proteins targeting of *C*. *pneumoniae* in host cells components with different parameters illustrates in Fig 2. The supplementary data provides details regarding proteins predicted to target nucleus of host cell during our analysis (S1 Table).

## Discussion

Epidemiological reports have founded several well-defined potential factors for the growth and development of cancer such as heredity, age, use of tobacco, diet, inflammation, and chronic infections with pathogens. Infection is the cause for approximately 16% of all malignancies worldwide [19]. A number of bacteria have shown the capacity to alter many pathways and molecules of host cells for their intracellular survival in the host. The thought that an infection of bacteria could promote to carcinogenesis disregards initially. However, landmark studies in the early 1990s established *C*. *pneumoniae* as a causative agent of different lung cancers, resulting in a new orientation of the scientific focus toward patterns of bacterial association with cancers (5, 6). *C*. *pneumoniae* bacterial derived effector molecules can change the internal environment of host cell through the production of chronic inflammation, inhibition of tumor suppressor mechanisms, induction of immunosuppression, and transformation of cells by transfer of oncogenes [7, 13, 14, 20–22]. The proteins of *C*. *pneumoniae* will be existed in the host cell during chronic infection and some proteins may migrate to the many organelles of the host cell such as nucleus, endoplasmic reticulum, Golgi apparatus, mitochondria etc. Proteins enter into host nuclei have many adverse effects that may inhibit or promote certain important biological activities leading to the development of cancer.

Subcellular protein targeting may be predicted by various tools, that works on different principles and different parameters. These parameters included composite motifs through the artificial neural feed-forward network, different binding grooves of importin α, simple Hidden Markov model, identity/alignment search, linear motifs and their role in cell signaling and regulation, support vector machine (SVM), and functional domain information along with sequential evolution information [23–28]. We predicted sub cellular proteins targeting using cNLS mapper, BaCeILo and Hum-mPLoc 2.0 predictors in order to achieve more accurate and consistent results.

Nuclear localization signals (NLSs) are very critical to ensure the selective transport of proteins into the nucleus [29]. The cNLS Mapper tool correctly locates the nuclear localization signals (NLSs) particularly to the importin α/β pathway by predicting NLS scores. The calculated NLSs are divided into two classes, monopartite (1 basic cluster) and bipartite (2 basic cluster) NLSs, according to the existence of cluster of rich basic amino acid residues. Furthermore, the scores of NLS are evaluated with four classes of profiles with specified cut off values. Higher values of NLS score show more NLS activities. Proteins with cut off value 8–10 were predicted as localized to the nucleus, 8–10 as partially in the nucleus, 3–5 as both in nucleus and cytoplasm, and 1–2 as particularly in cytoplasm. The proteins of *C*. *pneumoniae* indicating intermediary cut off value was included in a specific range of cut off value in protein list such as a cut off value 7.5 or above was rounded up to 8 while 7.4 or below was rounded down to 7. cNLS mapper was used to compute NLS activity instead of NLS sequence because NLS sequences are not stringent enough [26]. However, it must be remembered that the NLS profiles of cNLS mapper were produced by nuclear import assays using the data of yeast. Therefore, the prediction of NLS for other species may not be as accurate as in yeast although the importin α/β pathway of eukaryotes is highly conserved. Recent study identified a new bacterial protein SINC that targets the nuclear envelope in the infected and non-infected neighboring host cell with the potential of modifying nuclear envelope functions. These capabilities of *C*. *psittaci* bacteria may promote the process of destructive pathogenesis [30].

BaCelLo is a computational predictor used in our study for the subcellular localization of proteins in eukaryotes. Animals, plants, and fungi predictors were implemented in BaCelLo; hence, we used animal specific predictor. It is based on diverse SVMs for the prediction of nuclear, cytoplasmic, mitochondrial, secretory, and chloroplast targeting proteins [24]. BaCelLo predicts subcellular targeting based on residue sequence information and evolutionary information included in alignment profiles within the N and C termini and entire protein sequence.

Prediction of subcellular localization of proteins in human is a more challenging task. Another subcellular localization prediction tool Hum-mPLoc 2.0 was used to deal with the nuclear targeting of protein in human system. The Hum-mPLoc 2.0 tool predicts the proteins targeting on the basis of domain information and the sequential evolution information. The predictor computes 14 subcellular locations including nucleus, cytoplasm, mitochondrion, plasma membrane, endoplasmic reticulum, extracell, Golgi apparatus, cytoskeleton, endosome, lysosome, peroxisome, microsome, synapse, and centriole. Although the comparative results of BaCeILo and Hum-mPLoc 2.0 were demonstrated slight difference in subcellular localization of *C*. *pneumoniae* proteins in host organelles, the slight difference in results of BaCeILo and Hum-mPLoc 2.0 may be due to the existence of different data in their respective datasets used during prediction. Therefore, the little variations in outcomes obtained from different tools can be accordingly justified.

In this study, we present a systematic computational prediction of *C*. *pneumoniae* proteins using differently functioning predictors: NLS mapper, BaCeILo, and Hum-mPLoc 2.0 that works on different datasets. The prediction of BaCeILo based on animal dataset, whereas the Hum-mPLoc 2.0 worked on human specific datasets which includes 3,681 human proteins classified into 14 different human sub cellular locations. Therefore, the results were further narrow down and scrutinized after using human dataset specific predictor. It has been reported that many proteins can localize in the nucleus in the absence of NLS [24, 31]. Moreover, proteins less than 40kD can freely diffuse to nucleus [32]. According to our predictions, little variation in the results expected due to the use of different tools. Therefore, these results of *in silico* prediction require further experimental verification prior to any final conclusion. Furthermore, we focused on the potential effects of these nuclear targeting proteins in tumorigenesis and development of cancer.

### DNA replication and DNA binding proteins

The genomic instability is a crucial factor in cancer. Nonetheless, the mechanisms of its growth and development remain not fully understood. A frequently stated assumption is that anomalies in translesion DNA synthesis or error-prone phenotypes in DNA replication participate in genomic DNA instability and are prominent cause of the development of cancer. Such, erroneous DNA replication mechanisms have been implicated as an etiological factor in many cancers [33–35].

For instance, DNA polymerase beta protein is involved in approximate 30% all human tumors reported to date due to mutations [33, 34]. The bacterial DNA polymerase III subunit beta has a homolog of eukaryotic proliferating cell nuclear antigen (PCNA). PCNA is identified as a molecular marker for cell proliferation during replication [36, 37]. In our study, we found nuclear localization of DNA polymerase III subunit beta protein. PCNA was characterized as a potential antigen that is expressed during the phase of DNA synthesis in cell cycle and involved in carcinogenesis [38]. Therefore, during infection, the possible existence of two homologs proteins in same cell with unique enzymatic action alters the relative activity of host protein. As DNA polymerase III subunit beta is a DNA-replication connected proteins, the anomaly in DNA replication can also act as a factor for the growth and development of cancer.

Another chaperone protein DnaJ a homolog of HSP40 is also predicted as nuclear target that may change the activity of HSP40 and involved in carcinogenesis [39, 40]. In addition, certain DNA replication and binding proteins are also predicted as nuclear targeting proteins such as DNA gyrase subunit A, single-stranded DNA-binding protein (SSB), and primosomal protein, which may also be involved in the development of cancer.

### Gene expression associated proteins

Translational regulation is a crucial process in the progression and development of cancer. It manages both the overall expression of protein synthesis and the specific translation of selective mRNAs that may support various oncogenic properties including cell transformation, tumor cell survival, invasion, metastasis, and angiogenesis. The nuclear targeting of these gene expression proteins implies their potential roles in the growth and development of lung cancer. Consequently, alteration in the gene expression is connected with the growth and development of cancer through the dysregulation of many critical genes. Dysregulation may direct the activation of proto-oncogenes and suppression of anti-oncogenes [41].

The results of our study show that DNA-directed RNA polymerase β and β’ subunits of *C*. *pneumoniae* are targeted to host cell nucleus. This is consistent with other reports that demonstrated an alteration in the levels of gene expression in many hosts, including human and other eukaryotes, as an action of bacterial RNA polymerase. For instance, various human genes may be transcribed through the involvement bacterial transcription regulators using *E*. *coli* DNA-directed RNA polymerase II [42, 43]. These predicted transcription-associated proteins may efficiently bind to host DNA and consequently hinder the binding affinity of the host transcription regulators and ultimately deregulate gene expression [42]. Although it has been confirmed that *C*. *pneumoniae* is associated with alteration of host gene expression [21], its involvement in the progression and development of lung cancer in human requires further experimental assessment. Our result encompasses important findings that can contribute to this emerging field.

### DNA damage and repair proteins

Previous study showed that *C*. *pneumoniae* has the ability to induce DNA damage through the induction of reactive oxygen species (ROS) [13]. Our findings have demonstrated nuclear targeting of DNA-damaging proteins including exonuclease V subunit RecB, ribonuclease R, exodeoxyribonuclease VII small subunit, and exodeoxyribonuclease VII large subunit. Moreover, it is found that DNA mismatch repair is essential for enhancing the fidelity of replication in most of the organisms including bacteria, yeast, and humans etc. MutS has been identified as a protein of the ABC ATPase superfamily, which is involved in unpaired and mispaired bases in double stranded DNA that initiates mismatch repair. Mutation in MutS may be a possible cause of the growth and development of cancer [44, 45]. We predicted nuclear localization of DNA mismatch repair proteins MutS and MutL during the analysis. Alteration in mismatch repair proteins is potentially associated with various types of human cancers including lung.

## Conclusion

We proposed a new and integrative *in silico* approach for identifying the suspicious role of *C*. *pneumoniae* proteins in the growth and development of lung cancer in human. The results of *in silico* prediction revealed 47 candidates proteins. Out of which, various proteins may have the potential to trigger cancer growth through the alteration in replication, transcription, and DNA damage repair mechanism. It is confirmed that various proteins of *C*. *pneumoniae* can target to different organelles including nucleus and other parts of host cells, which may be an etiological cause of lung cancer. Our prediction data demonstrated more accuracy of computational prediction due to the use of different prediction tool based on different datasets, which may suggest that nuclear targeting proteins of *C*. *pneumoniae* can be potential targets for lung cancer management. Therefore, the outcome of this *in silico* study can open the new avenue for lung cancer research. Although the oncogenic potential and significant contribution of this nuclear-targeted protein of *C*. *pneumoniae* in growth and development of cancer was suggested by our knowledge and computational analysis, the confirmatory roles and specificity of these predicted proteins in carcinogenesis process require further experimental validation.

## Materials and Methods

### Selection of protein database

*C*. *pneumoniae* is an obligate intracellular gram negative pathogen, infects humans and suspiciously involved as an etiological agent of lung cancer [5, 46]. The proteome of *C*. *pneumoniae* TW-183 were downloaded from Uniprot database. Five proteomes of different strains of *C*. *pneumoniae* were available [15–18]. The proteome of TW-183 strain of *C*. *pneumoniae* was analyzed for the prediction of the nuclear localization signal and human cell subcellular localization using different computational tools.

### Prediction of nuclear localization signal

cNLS mapper tool for eukaryotic cells was used for the prediction of nuclear localization signal in TW-183 protein of *C*. *pneumoniae* [23]. The complete sequence of each *C*. *pneumoniae* protein was used for the prediction of monopartite and bipartite NLS sequence.

### Prediction of subcellular localization in eukaryotic cell organelles

The balanced subcellular localization predictor (BaCeILo) was used to predict the subcellular localization of TW-183 protein of *C*. *pneumoniae* in eukaryotic cell compartments. BaCelLo has based on three specific predictors for eukaryotic kingdoms including animals, plants, and fungi [24]. BaCeILo predict five classes of sub cellular localization including nuclear, mitochondrial, cytoplasmic, secretory, and chloroplast. We were done the prediction with animal specific predictor using proteins of TW-183 strain of *C*. *pneumoniae*.

### Prediction of subcellular localization in human cell organelles

Furthermore, human protein subcellular localization Hum-mPLoc 2.0 (Hum-mPLoc 2.0) predictor was used to predict the subcellular localization of TW-183 of *C*. *pneumoniae* proteins in nucleus and other cell organelles in human [25]. Hum-mPLoc 2.0 predict the fourteen classes of subcellular localization that includes nucleus, cytoplasm, mitochondrion, endoplasmic reticulum, centriole, cytoskeleton, endosome, extracell, Golgi apparatus, lysosome, microsome, peroxisome, plasma membrane, and synapse.

### Synchronization of BaCeILo predicted proteins with Hum-mPLoc 2.0 predictor

Moreover synchronization was preformed for the predication of nuclear targeting protein in human using Hum-mPLoc 2.0. The results of BaCelLo was used to narrow down the sub cellular localization of *C*. *pneumoniae* proteins.

## Supporting Information

S1 Table S1 Table provides details information of predicted proteins targeted to nucleus of host cells. (DOC)
