# Supplementary material for: Systems Biology Approaches for the Prediction of Possible Role of Chlamydia pneumoniae Proteins in the Etiology of Lung Cancer
Source: PLoS One. 2016 Feb 12;11(2):e0148530. doi: 10.1371/journal.pone.0148530 (PMC4752481; doi:10.1371/journal.pone.0148530)
Supplement: S1 Table — (DOC) [file pone.0148530.s001.doc]

**Table S1.** Details of *C. pneumoniae*  proteins targeting to the host cell nucleus as predicted by BaCeILo and Hum-mPLoc 2.0 (Proteins are arranged as per their monopartite and bipartite NLS cutoff value)

| **Sr No** | **A. number** | **Protein Name** | **Amino Acids** | **Function in bacteria** | **Protein Existence** | **pI** | **Mol Wt** | **NLS Mapper**  **Mono Bi NLS NLS** | | **BaCeILo & Hum-mPLoc 2.0** |
| --- | --- | --- | --- | --- | --- | --- | --- | --- | --- | --- |
| 1 | Q9Z8Z1 | Arginine repressor | 147 | Amino-acid biosynthesis, Arginine biosynthesis, Transcription, Transcription regulation | Inferred by homology | 8.82 | 16,024 | 0 | 3 | Nucleus |
| 2 | Q9Z8F1 | Deoxycytidine triphosphate deaminase | 190 | dCTP deaminase activity, pyrimidine ribonucleotide biosynthetic process | Inferred by homology | 5.13 | 21,475 | 0 | 2.3 | Nucleus |
| 3 | Q9Z9E9 | Chaperone protein DnaJ | 392 | ATP binding, DNA replication initiation, Stress response | Inferred by homology | 7.04 | 42,109 | 0 | 0 | Nucleus |
| 4 | Q9Z8K0 | DNA polymerase III subunit beta | 366 | 3'-5' exonuclease activity, DNA-directed DNA polymerase, Nucleotidyltransferase, Transferase, DNA replication | Inferred by homology | 4.95 | 40,365 | 0 | 0 | Nucleus |
| 5 | Q9K1Y4 | Exodeoxyribonuclease 7 small subunit | 75 | Exonuclease, Hydrolase, Nuclease, exodeoxyribonuclease VII activity | Inferred by homology | 4.55 | 8,809 | 0 | 0 | Nucleus |
| 6 | Q9Z8R4 | DNA gyrase subunit A | 834 | Isomerase, Topoisomerase, DNA topoisomerase type II (ATP-hydrolyzing) activity, DNA-dependent DNA replication | Inferred by homology | 6.62 | 93,839 | 0 | 6.8 | Nucleus |
| 7 | Q9Z874 | Threonylcarbamoyladenosine tRNA methylthiotransferase MtaB | 421 | Transferase, tRNA processing | Inferred by homology | 6 | 47,190 | 0 | 4.2 | Nucleus |
| 8 | Q9Z6Y2 | Primosomal protein N'  (ATP-dependent helicase PriA) | 749 | Helicase, Hydrolase, DNA replication, synthesis of RNA primer | Inferred by homology | 9.34 | 83,448 | 4.5 | 6.5 | Nucleus |
| 9 | Q9Z7G7 | RecBCD enzyme subunit RecB (Exonuclease V subunit RecB) | 1050 | Antiviral defense, DNA damage, DNA repair, exodeoxyribonuclease V activity | Inferred by homology | 6.23 | 121,056 | 0 | 5.4 | Nucleus |
| 10 | Q9Z969 | 50S ribosomal protein L31 type B | 109 | Ribonucleoprotein, translation | Inferred by homology | 9.59 | 12,404 | 0 | 5.3 | Nucleus |
| 11 | Q9Z848 | Ribonuclease R (RNase R) | 676 | Exonuclease, Hydrolase, Nuclease, exoribonuclease II activity | Inferred by homology | 8.93 | 76,354 | 0 | 7.1 | Nucleus |
| 12 | Q9Z9A0 | DNA-directed RNA polymerase subunit beta | 1252 | Nucleotidyltransferase, Transferase, Transcription | Inferred by homology | 5.57 | 140,110 | 7.5 | 4.6 | Nucleus |
| 13 | Q9Z999 | DNA-directed RNA polymerase subunit beta' | 1393 | Nucleotidyltransferase, Transferase, Transcription | Inferred by homology | 6.35 | 194,901 | 3.5 | 6.5 | Nucleus |
| 14 | Q9Z7K6 | Ribosome-recycling factor (RRF) (Ribosome-releasing factor) | 180 | Protein biosynthesis, translational termination | Inferred by homology | 8.44 | 20,169 | 0 | 5.3 | Nucleus |
| 15 | Q9Z8C2 | Ribosomal RNA small subunit methyltransferase H | 297 | Methyltransferase, Transferase, rRNA (cytosine-N4-)-methyltransferase activity | Inferred by homology | 8.27 | 33,839 | 0 | 5.6 | Nucleus |
| 16 | Q9Z765 | Protein translocase subunit SecA | 970 | Protein transport, Translocation, Transport | Inferred by homology | 5.79 | 111,092 | 0 | 6 | Nucleus |
| 17 | Q9Z9F7 | Tyrosine recombinase XerC | 312 | chromosome segregation, DNA integration, DNA recombination | Inferred by homology | 9.84 | 35,852 | 3 | 5.8 | Nucleus |
| 18 | Q9Z960 | Uncharacterized protein | 97 | Unknown | Inferred by homology | 9.43 | 11,098 | 0 | 6.2 | Nucleus |
| 19 | Q9Z8Z4 | Uncharacterized protein | 452 | Unknown | Protein predicted | 5.51 | 50,178 | 0 | 7.6 | Nucleus |
| 20 | Q9Z8C1 | Uncharacterized protein | 181 | Unknown | Inferred by homology | 5.01 | 20,863 | 0 | 3.8 | Nucleus |
| 21 | Q9Z7Z2 | Uncharacterized protein | 178 | Unknown | Inferred by homology | 8.6 | 20,682 | 2 | 4.8 | Nucleus |
| 22 | Q9Z7P6 | Uncharacterized protein | 238 | Unknown | inferred by homology | 5.07 | 27,370 | 0 | 3.5 | Nucleus |
| 23 | Q9Z7D7 | DNA topoisomerase 1 | 871 | Releases the supercoiling and torsional tension of DNA | inferred by homology | 8.64 | 98,892 | 3.5 | 5.5 | Nucleus |
| 24 | Q9Z7K3 | Outer membrane secretion protein Q | 919 | protein secretion | inferred by homology | 6.02 | 100,392 | 3.5 | 6.3 | Nucleus |
| 25 | Q9Z8I6 | Uncharacterized protein | 283 | Unknown | inferred by homology | 5.64 | 32,507 | 0 | 3.8 | Nucleus |
| 26 | Q9Z6Q5 | Uncharacterized protein | 173 | Unknown | Protein predicted | 5.51 | 19,248 | 0 | 2.6 | Nucleus |
| 27 | Q9JS82 | Uncharacterized protein | 368 | Unknown | Protein predicted | 7.93 | 42,766 | 0 | 4.6 | Nucleus |
| 28 | Q9Z8J3 | Uncharacterized protein | 168 | Unknown | Protein predicted | 9.93 | 19,099 | 10.5 | 3.5 | Nucleus |
| 29 | Q9K260 | Uncharacterized protein (YecA) | 171 | Unknown | Protein predicted | 6.01 | 19,411 | 0 | 5.6 | Nucleus |
| 30 | Q9Z7M8 | Uncharacterized protein | 389 | Unknown | Protein predicted | 4.67 | 43,403 | 0 | 5.4 | Nucleus |
| 31 | Q9Z6T1 | Uncharacterized protein | 343 | Unknown | Protein predicted | 6.38 | 39,665 | 7 | 5.2 | Nucleus |
| 32 | Q9Z9B9 | Uncharacterized protein | 461 | Unknown | Protein predicted | 10.06 | 50,823 | 5.5 | 6.6 | Nucleus |
| 33 | Q9Z7X7 | Uncharacterized protein | 87 | Unknown | Protein predicted | 9.89 | 9,880 | 0 | 3.2 | Nucleus |
| 34 | Q9Z8H3 | Uncharacterized protein | 371 | Unknown | Protein predicted | 8.27 | 40,571 | 6 | 3 | Nucleus |
| 35 | Q9Z9F5 | Uncharacterized protein | 288 | Unknown | Protein predicted | 9.82 | 31,901 | 7.5 | 9 | Nucleus |
| 36 | Q9Z8V9 | Uncharacterized protein | 223 | Unknown | Protein predicted | 9.17 | 24,113 | 2 | 5.7 | Nucleus |
| 37 | Q9Z6N9 | Uncharacterized protein | 510 | Unknown | Protein predicted | 8.45 | 57,917 | 0 | 4.9 | Nucleus |
| 38 | Q9Z6I9 | Uncharacterized protein | 456 | Unknown | Protein predicted | 5.45 | 50,112 | 0 | 4.4 | Nucleus |
| 39 | Q9Z727 | Metal dependent hydrolase | 265 | hydrolase activity | Protein predicted | 5.74 | 29,566 | 0 | 5.9 | Nucleus |
| 40 | Q9Z6T2 | Uncharacterized protein | 344 | Unknown | Protein predicted | 6.46 | 39,603 | 0 | 2.9 | Nucleus |
| 41 | Q7VQ96 | Uncharacterized protein | 416 | Unknown | Protein predicted | 6.82 | 46,042 | 0 | 4.6 | Nucleus |
| 42 | Q7VQ18 | Uncharacterized protein | 146 | Unknown | Protein predicted | 9.16 | 16,479 | 0 | 5 | Nucleus |
| 43 | Q7VQ73 | Uncharacterized protein | 195 | Unknown | Protein predicted | 7.59 | 23,037 | 4.5 | 5.2 | Nucleus |
| 44 | Q7VPW6 | YwbM | 382 | Unknown | Protein predicted | 7.15 | 40,616 | 0 | 3.4 | Nucleus |
| 45 | Q7VPW4 | Uncharacterized protein | 204 | hydrolase activity | Protein predicted | 9.44 | 23,665 | 0 | 4.2 | Nucleus |
| 46 | Q7VQ77 | Uncharacterized protein | 340 | Unknown | Protein predicted | 6.66 | 38,528 | 0 | 3.1 | Nucleus |
| 47 | Q7VPY9 | Uncharacterized protein | 359 | Unknown | Protein predicted | 6.16 | 40,011 | 0 | 4.3 | Nucleus |
